# Kazakhstani Drivers and Substance Abuse During COVID-19: A Study of Patterns and Disaster Readiness

**DOI:** 10.3390/healthcare13070756

**Published:** 2025-03-28

**Authors:** Assiya Kussainova, Almas Kussainov, Laura Kassym, Yerbolat Baikenov, Dana Kozhakhmetova, Dinara Mukanova, Saltanat Adilgozhina, Ainash Orazalina, Yerbol Smail

**Affiliations:** 1 Department of General Medical Practice with a Course of Evidence-Based Medicine, NJSC Astana Medical University, Astana 010000, Kazakhstan; kussainova.as@amu.kz (A.K.); kassym.l@amu.kz (L.K.); 2 Department of Psychiatry and Narcology, NJSC Astana Medical University, Astana 010000, Kazakhstan; 3 Project Implementation Department, Republican Medical Institute, Astana 010000, Kazakhstan; rmi_2015@mail.ru; 4 Department of Internal Medicine and Rheumatology, NJSC Semey Medical University, Semey 071400, Kazakhstan; dana.kozhakhmetova@smu.edu.kz; 5 Department of Simulation and Educational Technologies, NJSC Semey Medical University, Semey 071400, Kazakhstan; dinara.mukanova@smu.edu.kz; 6 Department of Family Medicine, NJSC Semey Medical University, Semey 071400, Kazakhstan; saltanat.adilgozhina@smu.edu.kz; 7 Department of Molecular Biology and Medical Genetics Named After the Academician of the National Academy of Sciences Republic of Kazakhstan Raissov T.K., NJSC Semey Medical University, Semey 071400, Kazakhstan; ainash.orazalina@smu.edu.kz; 8 Department of Infectious Diseases, Dermatovenereology and Immunology, NJSC Semey Medical University, Semey 071400, Kazakhstan; erbol.smail@smu.edu.kz

**Keywords:** addictions, pandemic, COVID-19, alcohol, cannabinoids, opioids, driver

## Abstract

**Background/Objectives**: The COVID-19 pandemic has significantly affected public health and social behavior, contributing to increased psychoactive substance (PAS) use due to social isolation, economic stress, and uncertainty. This study aims to assess the impact of the pandemic on alcohol, cannabinoid, and opioid consumption among drivers involved in road traffic accidents (RTAs) in Kazakhstan. Understanding these patterns is essential for improving public health policies and road safety measures during crises. **Methods**: This retrospective cross-sectional study analyzed medical records from the Digital System of Medical Examination, a national database of drivers involved in traffic accidents in Kazakhstan. This study included 157,490 anonymized records from 1 January 2019, to 31 December 2020, categorizing cases into pre-COVID-19 and COVID-19 groups on the basis of the first nationwide lockdown on 16 March 2020. Statistical analyses, including prevalence rates and relative changes, were conducted via SPSS 20, while spatial distributions were visualized via QGIS software. **Results**: An analysis of all the records revealed a 12.9% decline in traffic accidents during the pandemic, with male drivers predominating during both periods. The mean age of the drivers in the compared groups was 36. Alcohol and cannabinoid use significantly increased during the COVID-19 period by 3.71% and 11.51%, respectively. In contrast, opioid use declined by 10.00%, but the difference was not statistically significant. The greatest increase in positive alcohol tests among drivers was observed in the Atyrau (94.80%), Pavlodar (35.43%), and North Kazakhstan (31.02%) regions, and Atyrau also presented the greatest increase in cannabinoid-positive cases. **Conclusions**: The results indicate that the COVID-19 pandemic and related lockdown measures have affected PAS consumption patterns among drivers. These findings are crucial for informing policies and developing strategies to improve road safety during future public health emergencies.

## 1. Introduction

The coronavirus disease 2019 (COVID-19) pandemic has had profound global consequences, impacting public health, economic stability, and social behavior [1]. To mitigate the spread of infection, governments worldwide have implemented various restrictive measures, including mandatory mask-wearing, social distancing, and quarantine isolation [2]. These efforts significantly limit access to essential activities, such as education, employment, mobility, and public events [3]. Several studies have concluded that these circumstances lead to clinical and psychological issues, adversely affecting the wellness and health of the population [4]. For example, a systematic review by Vindegaard et al. (2020) revealed lower psychological well-being and higher levels of anxiety and depression among the population than in the pre-COVID-19 period [5].

Social isolation and associated emotional distress may have contributed to increased alcohol consumption [6] and the use of other psychoactive substances (PASs), particularly cannabinoids and opioids [7]. For example, in 2020, the European Monitoring Center for Drugs and Drug Addiction recorded alterations in alcohol and illegal drug usage patterns, implying that these changes were related to the worldwide lockdown situation [8]. Furthermore, rising unemployment rates, financial difficulties, poor living conditions, and uncertainty about the future may have exacerbated substance use [9]. The prolonged nature of the pandemic, coupled with increased PAS use, has contributed to an increase in traffic violations and a greater proportion of severe road traffic accidents (RTAs) with fatal outcomes [10]. Zuo et al. (2020) reported an increase in the traffic accident mortality rate, which increased from 1.4 to 1.9 per 1000 crashes during the first three weeks of April 2020, as compared with the corresponding period in February 2020 [11]. Additionally, a study by the National Highway Traffic Safety Administration (NHTSA) reported an increase in driving under the influence of alcohol and drugs from March to July 2020 [12]. In contrast, a study by Hostiuc et al. (2021) reported a significant reduction in cases of driving under the effect of alcohol during the first wave of the pandemic [13].

In March 2020, nationwide quarantine measures were introduced in Kazakhstan to prevent the spread of COVID-19 [14]. These restrictions affected transportation systems and population mobility, leading to a decline in overall traffic volume. Between January and May 2020, the number of criminal traffic offenses decreased by 33% as compared with offenses during the same period in the previous year. However, statistical data indicate that 88.8% of all individuals held accountable for traffic violations were charged with driving under the effect of alcohol or drugs [15]. According to national analysts, alcohol sales in Kazakhstan increased by 11% during the pandemic, whereas in 2019, this growth was only 2%. This surge in alcohol consumption was attributed to lockdown restrictions [16]. Additionally, the closure of borders with neighboring countries reduced the availability of opioid (heroin) and cannabis-based (marijuana, hashish) drugs on the market, leading to increased popularity of synthetic narcotics distributed via the internet [17].

To date, research on substance use among drivers remains significantly limited, restricting a comprehensive understanding of its prevalence and trends, particularly during major public health crises. Examining the relationship between the COVID-19 pandemic and driving under the influence of alcohol and/or drugs is essential for informing public health policies and enhancing road safety measures in crisis situations. Analyzing substance use patterns in traffic accidents during a pandemic can offer valuable insights into the impact of external factors on high-risk behaviors and highlight potential areas for policy improvement [18].

The hypothesis of this study is that the COVID-19 pandemic has had a significant effect on the frequency and nature of RTAs involving PAS use in Kazakhstan. This study aimed to assess the effects of the pandemic on the consumption of alcohol, cannabinoids, and opioids among drivers involved in RTAs in Kazakhstan.

## 2. Materials and Methods

### 2.1. The Database

For this retrospective study, we analyzed medical records obtained from the Digital System of Medical Examination, a national database containing the records of drivers involved in traffic accidents in Kazakhstan. The Republican Medical Institute (Astana, Kazakhstan) has been responsible for creating, developing, and maintaining the database since 2016. During the study period, the Digital System of Medical Examination was implemented across 18 regions, encompassing 176 medical institutions including regional centers for mental health, central district hospitals, and rural clinics. Only 13 regions and one city were covered by the mentioned system’s service, as other regions and cities with populations exceeding one million have their own medical examination systems.

The database records are maintained in accordance with Order No. 2023/2020 issued by the Minister of Healthcare of the Republic of Kazakhstan on 25 November 2020 [19]. The record can be completed only if all the data are fully described. The Digital System of Medical Examination is designed to digitize the collection and storage of medical examination data to determine the use of psychoactive substances and the state of intoxication. The system has undergone the necessary testing by the State Technical Service of the National Security Committee of the Republic of Kazakhstan and meets all the information security requirements. The system enables the centralization of medical examination procedures and ensures monitoring at both the regional and national levels. Access to the system is granted only to medical professionals conducting medical examinations and employees of analytical departments. Authorization is performed via a login and password, with access restricted to workplace computers only.

This database provides the most up-to-date nationwide epidemiology data on demographic and clinical characteristics and the results and details of laboratory tests on psychoactive substance use in drivers from 13 regions and one city. In our research, substance use refers to the consumption of psychoactive substances, including alcohol and illicit substances, which may alter an individual’s mental or physical state [20]. Blood samples for the detection of alcohol or drug use are taken from drivers by police request after they have been involved in road traffic accidents.

### 2.2. Study Design and Proceedings

This was a retrospective cross-sectional study. We extracted 157,490 anonymized records from the database of drivers who were involved in road traffic accidents and of whom driving under the influence of substances was confirmed. The time period was between 1 January 2019, and 31 December 2020. The Kazakhstan government imposed the first lockdown on 16 March 2020 [14], and this date was used as a reference point to divide all records into pre-COVID-19 and COVID-19 groups. A retrospective study design was optimal in this case, as it allows researchers to analyze a large dataset efficiently, assess the impact of an external event (COVID-19 lockdown), and draw meaningful conclusions from already collected data.

### 2.3. Definitions Used

PAs are chemical compounds of either natural or synthetic origin that affect the central nervous system, leading to alterations in emotional state, cognitive function, and consciousness. Their use can modify sensory perception, emotional responses, and reactions to external stimuli [20]. The primary categories of psychoactive substances include alcohol, cannabinoids, and opioids [21].

Alcohol is a colorless, volatile, and flammable liquid formed through the natural fermentation of sugars. It serves as the primary psychoactive component in beverages such as wine, beer, spirits, and other alcoholic drinks [22].

Cannabinoids (such as marijuana, hashish, and anasha) are a group of chemical compounds of either synthetic or natural origin that exhibit psychotropic effects [23].

Opioids are substances that can bind to opioid receptors in the body and are located primarily in the central nervous system and the gastrointestinal tract. They are classified into natural opioids (such as opium, morphine, and codeine), semisynthetic opioids (including heroin, oxycodone, and hydromorphone), and synthetic opioids, which are synthesized entirely in laboratories [24].

### 2.4. Statistical Analysis

The prevalence rates, along with 95% confidence intervals, per 100 tested drivers were presented by region to evaluate the burden of alcohol or substance use. For the purposes of time series analysis, the percentage of relative changes in cases from the pre-COVID-19 period to the COVID-19 period was calculated via the following formula:

$$\mathrm{Percentage}\;\mathrm{of}\;\mathrm{relative}\;\mathrm{changes}\;\mathrm{in}\;\mathrm{cases}=\frac{{cases}_{COVID-19}-{cases}_{pre-COVID-19}}{{pre-}_{COVID-19}}\times 100\%$$

In addition, the chi-square test was employed to assess differences in the prevalence of substance use among drivers across various regions during the COVID-19 period as compared with the pre-COVID-19 period. Statistical significance was set at *p* < 0.05.

All the statistical analyses were performed via SPSS 20 software. To enable better visualization of the spatial distribution of driving under substances, administrative country maps were constructed with the help of QGIS software (version 3.28.3 Firenze) [25]. The severity of the burden of alcohol or substance use among drivers has not been clearly defined in the literature [26]. In this paper, a geographical area with more incident cases than other areas, as visualized by software, is defined.

### 2.5. Ethics Statement

This study was approved by the Ethics Committee of Astana Medical University (Approval #15; dated 21 October 2021) within the guidelines established by the 1964 Declaration of Helsinki.

## 3. Results

In our study, we analyzed 157,490 records registered in a national database, including 97,413 (56.47%) of those who were tested in the pre-COVID-19 period and 60,076 (43.53%) of those who passed medical examinations during the pandemic. The male/female ratio in both periods was 9:1, and the mean age of the drivers in the compared groups was 36. The vast majority of the subjects (n = 119,180; 75.67%) were in the 18–44 years old age group (Table 1).

Key findings indicate a statistically significant increase in alcohol and cannabinoid use during the COVID-19 period, with percentage changes of 3.71% and 11.51%, respectively, and *p* values of 0.001 and 0.010, respectively, confirming the significance of these trends. In contrast, opioid use showed a minor decline (−10.00%), with a nonsignificant *p* value of 0.547, suggesting no substantial shift in usage patterns (Table 2).

**Figure 1:**
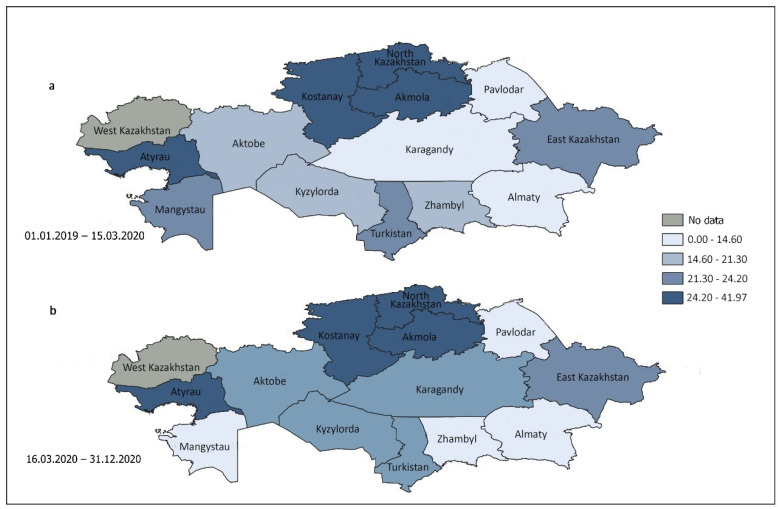
Republican trends in the prevalence of alcohol use among tested drivers in the pre-COVID-19 (**a**) and COVID-19 (**b**) periods in 13 regions and one city.

The current study investigated patterns of alcohol use among drivers who underwent testing in 13 Kazakhstani areas and one city during both the pre-COVID-19 and COVID-19 periods (Figure 1). The prevalence of positive tests for alcohol consumption in tested subjects is greater in regions with darker shades of blue on the map. The gray color indicates that data from this part of the republic are not available.

During the period of COVID-19 mobility restrictions, there was a 3.71% increase in alcohol consumption among participants, increasing from 16.98 cases per 100 subjects in the pre-COVID-19 period to 17.61 cases per 100 subjects within the country. During the COVID-19 restriction period, as compared with the pre-COVID-19 period, the prevalence of positive alcohol tests among drivers increased significantly in the Atyrau, Pavlodar, and North Kazakhstan regions, with the most notable percentage increases of these regions being 94.80%, 35.43%, and 31.02%, respectively. In contrast, this parameter declined substantially in the Aktobe, Mangystau, and Zhambyl regions, with the most significant decreases of these regions being −11.08%, −33.55%, and −28.39%, respectively (Table 3).

**Figure 2:**
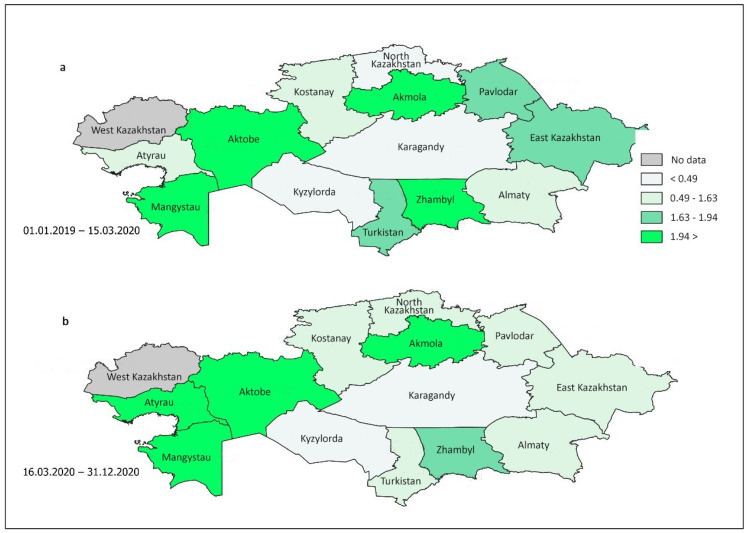
Republican trends in the prevalence of cannabinoid use among tested drivers during the pre-COVID-19 (**a**) and COVID-19 (**b**) periods in 13 regions and one city.

Figure 2 depicts the patterns of cannabinoid use among subjects tested at different locations in Kazakhstan, both before and during COVID-19 mobility restrictions. The darker shades of green on the map indicate higher levels of cannabinoid use among drivers in various regions. Notably, Figure 2 reveals significant variations in the prevalent cases of positive tests for cannabis and related products across different parts of Kazakhstan during both observation periods.

Notably, in the two western regions, there was a notable increase in marijuana usage among drivers. Specifically, in the Atyrau region, the number of prevalent cases increased from 0.89 (95% CI 0.70–1.14) to 2.20 (95% CI 1.64–2.96) during the comparison periods, whereas in both periods, the same parameter increased from 2.20 (95% CI 1.74–2.79) to 3.35 (95% CI 2.84–3.95) in Mangystau (Table 4). Additionally, the chi-square test indicated a significant increase in cannabis use in the Karaganda region (*p* = 0.028). In contrast, cannabis use declined in the Zhambyl, Turkestan, East Kazakhstan, Pavlodar, and North Kazakhstan regions during the COVID-19 period.

**Figure 3:**
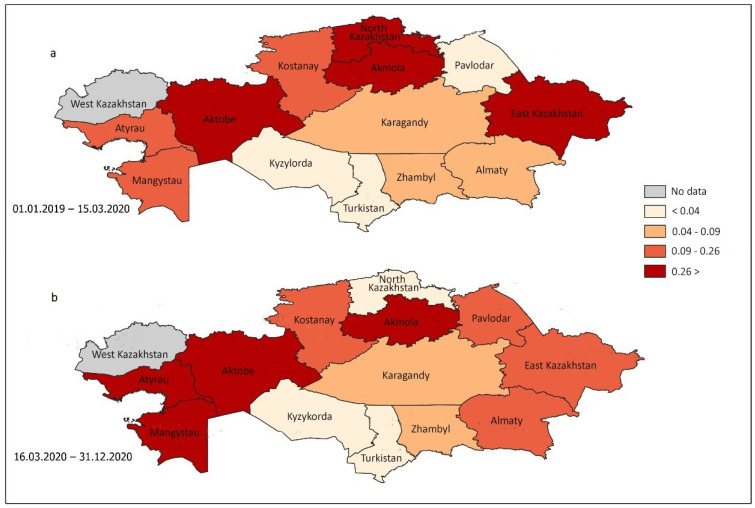
Republican trends in the prevalence of opioid use among tested drivers during the pre-COVID-19 (**a**) and COVID-19 (**b**) periods in 13 regions and one city.

Figure 3 illustrates the variations in opioid consumption among the tested individuals across different regions of Kazakhstan before and after the enforcement of mobility restrictions in response to the COVID-19 pandemic. Notably, the Pavlodar, Mangystau, Atyrau, and Almaty regions presented a substantial increase in the prevalence of positive opioid tests. Conversely, the North Kazakhstan and East Kazakhstan regions exhibited a decline in cannabinoid use among drivers during the COVID-19 period.

The examination of the data revealed no statistically significant difference in the consumption of opioids nationwide during the two periods under investigation. The prevalence of opioid use among drivers in the Pavlodar region increased significantly from 0.04 (95% CI 0.01–0.13) to 0.25 (95% CI 0.15–0.42), whereas in the Aktobe region, the prevalence of opioid use among drivers significantly decreased from 0.85 (95% CI 0.70–1.03) to 0.48 (95% CI 0.34–0.68) (Table 5). In addition, the chi-square test revealed a significant increase in opioid use in the Atyrau region and a significant decrease in the Aktobe region (*p* = 0.005 for both).

## 4. Discussion

The objective of our study was to assess the impact of the COVID-19 pandemic on the consumption of alcohol, cannabinoids, and opioids among drivers involved in RTAs in Kazakhstan. An analysis of 157,490 records from the national database Digital System of Medical Examination revealed that the total number of traffic accidents during the pandemic decreased by 12.9% as compared with the total number during the pre-pandemic period, with male drivers consistently outnumbering female drivers during both periods. The average age of traffic accident perpetrators was 36 years. These findings align with those of global research, which reported a decline in traffic accidents during the COVID-19 restrictions in the United States, Spain, Ireland, and England [27,28] largely attributed to reduced traffic volume. According to the U.S. National Highway Traffic Safety Administration, in 2019, men were 3.25 times more likely to be involved in traffic accidents because of speeding or driving under PAS [29]. The average age of accident participants varied but predominantly fell within the 18–40 age groups [30].

Our study further revealed that alcohol consumption in Kazakhstan increased by 3.71% during the pandemic as compared with consumption during 2019. Among the 13 regions examined, the highest frequency of positive alcohol test results among drivers was observed in the Atyrau, Pavlodar, and North Kazakhstan regions (94.80%, 35.43%, and 31.02%, respectively). Cannabinoid use rose by 11.51%, with a particularly notable increase in the two western regions of the country. Specifically, the prevalence of marijuana use in Atyrau increased from 0.89 to 2.20, whereas in Mangystau, it rose from 2.20 to 3.35. The analysis revealed no significant nationwide differences in opioid use between the study periods, but regional data revealed a higher prevalence among drivers in Pavlodar and Atyrau. When our findings are compared with global trends, the results appear inconsistent across different studies [12,31,32].

For example, Thomas et al. (2020) reported that the prevalence of PAS use among drivers significantly increased during the pandemic, with cannabinoid use being more common than alcohol consumption (32.7% vs. 28.3%), and the prevalence of opioid use doubled from 7.5% to 13.9% [31]. Similar results were reported in another large-scale study conducted among respondents in Canada and the United States, where PAS use among drivers increased during the pandemic as compared with the pre-pandemic period [12]. Conversely, a study conducted in Romania reported a significant decrease in the incidences of driving under the effect of alcohol during the first 6 months of lockdown [13]. The variations in alcohol and drug consumption trends across different regions of Kazakhstan may be attributed to several socioeconomic, cultural, and demographic factors. For example, the Atyrau region, located in western Kazakhstan, is a major center of the oil and gas industry and is characterized by higher income levels but also more demanding working conditions. Additionally, Atyrau’s proximity to the Russian border may facilitate illicit drug trafficking in the region [32].

Potential factors contributing to increased PAS use during the COVID-19 pandemic include social isolation and heightened stress and anxiety related to SARS-CoV-2 [3]. A study conducted in the United Kingdom demonstrated a general rise in mental health disorders among individuals aged 16 years and older in 2020 as compared with 2019 [33]. In Kazakhstan, an analysis by the public policy research center PaperLab revealed that one-third of young people in the Atyrau region experienced symptoms of depression during the pandemic, whereas only 1% of 513 respondents indicated that they were willing to seek professional help [34]. However, later longitudinal studies concluded that levels of depression and generalized anxiety disorder did not necessarily increase due to or during the early phase of the pandemic [35,36]. Therefore, other factors contributing to increased PAS use during the pandemic may include urbanization levels, substance availability, economic well-being, law enforcement measures, and preventive programs. For example, Kristianssen et al. (2018) reported that drug use was more prevalent among drivers in high-income countries, whereas alcohol remained the most common substance among drivers in low- and middle-income countries [37]. Limited access to psychiatric care due to quarantine restrictions may have particularly affected individuals with substance use disorders or mental health conditions, leading to an increase in illicit drug acquisition as a form of self-medication [38]. The Republican Scientific and Practical Center for Mental Health, located in the Pavlodar region, was the first to implement remote medical services during the pandemic, enabling physicians to conduct patient consultations online or via telephone. This initiative potentially streamlined the process of prescribing specialized medications [39]. Additionally, the region hosts an active methadone distribution center as part of the state-funded opioid substitution therapy program for individuals with opioid dependence [40].

Kazakhstan has faced several crises comparable to the COVID-19 pandemic in terms of their impacts on public health, the economy, and social life, although none have reached the same scale. Notable examples include the Asian financial crisis (1997–1998) [41], the H1N1 influenza pandemic in 2009 [42], the avian influenza outbreak in 2020 [43], the January 2022 civil unrest [44], and environmental crises such as floods and droughts in 2024 [45]. However, patterns of substance use during these emergencies in Kazakhstan have not been systematically examined, emphasizing a significant research gap that warrants further investigation. These crises illustrate the fact that Kazakhstan has repeatedly encountered severe challenges requiring emergency state intervention and societal adaptation.

Regulations on driving under such influence vary across countries. In Europe, BAC limits are typically 0.05% [46], whereas the U.S. sets it at 0.08% [47], with some European nations enforcing stricter thresholds of 0.02–0.03% [48]. Many European countries also apply a zero-tolerance policy for drugs [49]. Forces in Europe and the U.S. include breathalyzers, roadside drug tests, and random screenings [50]. In contrast, Russia allows a BAC of 0.03% [51], whereas Kazakhstan enforces a strict 0.00% limit [52]. Drug testing in Russia and Central Asia is less rigorous and often requires medical examination. Adopting Western enforcement practices could improve road safety in the region. In 2020, to mitigate traffic accidents related to alcohol and drug use, Kazakhstan increased penalties, extending driver license suspensions to 7 years and introducing administrative detention for up to 15 days. In cases involving casualties, offenders face imprisonment from 1 to 7 years [53]. National television networks regularly broadcast public awareness campaigns highlighting the risks of PAS use. Additionally, electronic emergency response systems utilizing GPS technology were developed and implemented, enabling automatic accident reporting to emergency services within 20 s [54].

Thus, the findings of our study support the hypothesis that the COVID-19 pandemic had a significant effect on the frequency and nature of RTAs associated with PAS use in Kazakhstan. However, we cannot establish a definitive causal relationship between the pandemic and increased PAS use. Furthermore, our dataset did not include data from Kazakhstan’s two largest cities, Astana and Almaty, owing to their autonomous medical examination databases and administrative restrictions. Additionally, a limitation of this study is the imbalance in sample size, with a substantially lower number of reported incidents in 2020. Nevertheless, this study represents the first large-scale investigation in Kazakhstan examining PAS use among drivers before and during the COVID-19 pandemic.

## 5. Conclusions

In summary, this study provides compelling evidence that the COVID-19 pandemic and the associated lockdown measures influenced the alcohol consumption patterns of vehicle drivers. The observed increase in alcohol-impaired driving during the lockdown period raises critical concerns about the potential for sustained alcohol misuse beyond the pandemic. Furthermore, the persistent use of PSAs, such as cannabinoids and opioids, despite quarantine restrictions and border closures, is particularly concerning. Consequently, road safety initiatives implemented during pandemics and future public health crises must be strategically designed to effectively communicate the risks associated with alcohol and drug consumption. Moving forward, a more comprehensive investigation into the underlying determinants of PSAs use—particularly in relation to mental health, socioeconomic factors, and the consumption of alcohol, cannabinoids, and opioids within the general population—is essential.

## Figures and Tables

**Table 1:** The main characteristics of drivers during the pre-pandemic and pandemic groups.

	Pre-COVID-19 Period	COVID-19 Period	Total
**Gender. n (%)**			
Male	88,941 (91.30)	55,218 (91.91)	144,159 (91.54)
Female	8473 (8.70)	4858 (8.09%)	13,331 (8.46)
**Age**			
M (SD)	36.17 (12.15)	36.10 (12.25)	36.14 (12.19)
Me (Q1-Q3)	33.00 (27.00–44.00)	33.00 (27.00–43.00)	33.00 (27.00–43.00)
**Age groups. n (%)**			
<18 yrs	976 (1.00)	738 (1.23)	1714 (1.09)
18–44 yrs	73,649 (75.60)	45,531 (75.79)	119,180 (75.67)
45–59 yrs	17,611 (18.08)	10,458 (17.41)	28,069 (17.82)
60–74 yrs	4919 (5.05)	3167 (5.27)	8086 (5.13)
75–90 yrs	259 (0.27)	181 (0.30)	440 (0.28)
>90 yrs	0 (0.0)	1 (0.00)	1 (0.00)

**Table 2:** Prevalent cases of positive results for alcohol or substance use among tested drivers during the pre-COVID-19 and COVID-19 periods.

Prevalent Cases, ×10^2^ (95% CI)						
	Pre-COVID-19 Period	COVID-19 Period	Rate Difference (95% CI)	Percentage Change (%)	Chi-Square Test	*p* Value
Alcohol	16.98 (16.75–17.22)	17.61 (17.31–17.92)	−0.63 (−1.01 to −0.24)	3.71	10.417	0.001
Cannabinoids	1.39 (1.32–1.47)	1.55 (1.45–1.65)	−0.16 (−0.285 to −0.03)	11.51	6.633	0.010
Opioids	0.20 (0.17–0.23)	0.18 (0.15–0.22)	0.01 (−0.03 to 0.05)	−10.00	0.363	0.547

**Table 3:** Prevalent cases of positive alcohol use among tested drivers in the pre-COVID-19 and COVID-19 periods depending on region.

Region	Incident Cases. ×10 ^2^ (95% CI)					
	Pre-COVID-19	COVID-19	Rate Difference (95% CI)	Percentage Change (%)	Chi-Square Test	*p* Value
Shymkent City	6.10 (5.73–6.49)	6.82 (6.32–7.35)	−0.72 (−1.37 to −0.08)	11.80	4.995	0.025
Aqmola region	35.66 (31.59–39.94)	32.94 (30.43–35.56)	2.71 (−2.22 to 7.78)	−7.63	1.189	0.275
Aktobe region	20.84 (20.11–21.59)	18.53 (17.60–19.49)	2.30 (1.08 to 3.51)	−11.08	13.800	0.000
Almaty region	14.09 (13.15–15.08)	12.59 (10.30–15.31)	1.49 (−1.45 to 4.05)	−10.65	1.109	0.292
Atyrau region	24.42 (23.44–25.42)	47.57 (45.36–49.78)	−23.14 (−25.59 to −20.69)	94.80	397.688	0.000
East Kazakhstan region	22.46 (21.79–23.14)	22.06 (21.10–23.05)	0.40 (−0.80 to 1.58)	−1.78	0.439	0.508
Zhambyl region	16.10 (15.30–16.94)	11.53 (10.62–12.51)	4.56 (3.28 to 5.81)	−28.39	47.226	0.000
Karagandy region	14.05 (13.55–14.57)	15.14 (14.40–15.92)	−1.09 (−2.02 to 0.17)	7.76	5.571	0.018
Kostanay region	33.66 (32.06–35.30)	34.12 (32.50–35.78)	−0.46 (−2.79–1.86)	1.37	0.155	0.693
Kyzylorda region	18.37 (17.37–19.41)	20.60 (19.30–21.96)	−2.23 (−3.93 to −0.55)	12.14	6.973	0.008
Mangystau region	21.70 (20.27–23.20)	14.42 (13.38–15.54)	7.28 (5.44 to 9.13)	−33.55	63.682	0.000
Pavlodar region	9.68 (8.93–10.49)	13.11 (12.36–13.91)	−3.43 (−4.53 to −2.31)	35.43	36.168	0.000
North Kazakhstan region	40.97 (36.81–45.27)	53.68 (48.66–58.64)	−12.71 (−19.34 to −5.93)	31.02	14.212	0.000
Turkestan region	23.58 (16.52–32.50)	20.13 (18.44–21.93)	3.45 (−4.23 to 13.03)	−14.63	0.744	0.388
Nationwide	16.98 (16.75–17.22)	17.61 (17.31–17.92)	−0.63 (−1.01 to −0.24)	3.71	10.417	0.001

**Table 4:** Prevalent cases of positive results of cannabinoid use among tested drivers during the pre-COVID-19 and COVID-19 periods depending on region.

Region	Incident Cases. ×10 ^2^ (95% CI)					
	Pre-COVID-19	COVID-19	Rate Difference (95% CI)	Percentage Change (%)	Chi-Square Test	*p* Value
Shymkent city	1.22 (1.06–1.41)	1.53 (1.30–1.80)	−0.30 (−0.62 to −0.001)	25.41	4.044	0.044
Aqmola region	2.59 (1.52–4.38)	2.64 (1.90–3.67)	−0.05 (−1.62 to 2.01)	1.93	0.004	0.951
Aktobe region	3.14 (2.84–3.48)	2.66 (2.30–3.09)	0.47 (−0.05 to 0.98)	−15.29	3.264	0.071
Almaty region	1.57 (1.26–1.96)	0.74 (0.32–1.72)	0.83 (−0.29 to 1.44)	−52.87	2.842	0.092
Atyrau region	0.89 (0.70–1.14)	2.20 (1.64–2.96)	−1.31 (−2.11 to −0.67)	147.19	22.794	0.0001
East Kazakhstan region	1.74 (1.54–1.97)	1.52 (1.26–1.84)	0.21 (−0.16 to 0.57)	−12.64	1.379	0.240
Zhambyl region	1.96 (1.67–2.29)	1.80 (1.45–2.24)	0.15 (−0.38 to 0.65)	−8.16	0.369	0.543
Karagandy region	0.27 (0.20–0.36)	0.44 (0.32–0.60)	−0.16 (−0.34 to −0.01)	62.96	4.812	0.028
Kostanay region	0.80 (0.54–1.16)	0.62 (0.40–0.96)	0.17 (−0.27 to 0.62)	−22.50	0.677	0.411
Kyzylorda region	0.13 (0.06–0.26)	0.20 (0.10–0.41)	−0.07 (−0.30 to 0.11)	53.85	0.696	0.404
Mangystau region	2.20 (1.74–2.79)	3.35 (2.84–3.95)	−1.15 (−1.92 to −0.35)	52.27	8.270	0.004
Pavlodar region	1.68 (1.37–2.05)	1.59 (1.33–1.91)	0.08 (−0.36 to 0.55)	−5.36	0.141	0.707
North Kazakhstan region	0.39 (0.11–1.40)	0.79 (0.27–2.30)	−0.40 (−2.12 to 0.90)	102.56	0.633	0.426
Turkestan region	1.89 (0.52–6.62)	1.48 (1.04–2.10)	0.40 (−1.28 to 5.85)	−21.69	0.113	0.737
Nationwide	1.39 (1.32–1.47)	1.55 (1.45–1.65)	−0.16 (−0.28 to −0.03)	11.51	6.633	0.010

**Table 5:** Prevalent cases of positive results of opioid use among tested drivers during the pre-COVID-19 and COVID-19 periods depending on region.

Region	Incident Cases. ×10 ^2^ (95% CI)					
	Pre-COVID-19	COVID-19	Rate Difference (95% CI)	Percentage Change (%)	Chi-Square Test	*p* Value
Shymkent city	0.01 (0.00–0.05)	0.03 (0.01–0.09)	−0.02 (−0.09 to 0.01)	200.00	2.297	0.130
Aqmola region	1.00 (0.43–2.31)	0.62 (0.32–1.22)	0.37 (−0.53 to 1.86)	−38.00	0.702	0.402
Aktobe region	0.85 (0.70–1.03)	0.48 (0.34–0.68)	0.36 (0.10–0.60)	−43.53	7.879	0.005
Almaty region	0.06 (0.02–0.18)	0.15 (0.03–0.83)	−0.09 (−0.93 to 0.10)	150.00	0.662	0.416
Atyrau region	0.11 (0.06–0.22)	0.41 (0.21–0.81)	−0.30 (−0.73 to −0.05)	272.73	7.991	0.005
East Kazakhstan region	0.27 (0.20–0.37)	0.14 (0.08–0.26)	0.12 (−0.02 to 0.24)	−48.15	3.183	0.074
Zhambyl region	0.05 (0.02–0.13)	0.05 (0.01–0.17)	0.006 (−0.13 to 0.10)	0.00	0.022	0.882
Karagandy region	0.08 (0.05–0.14)	0.05 (0.02–0.12)	0.03 (−0.05 to 0.10)	−37.50	1.120	0.290
Kostanay region	0.15 (0.05–0.36)	0.19 (0.09–0.41)	−0.03 (−0.29 to 0.21)	26.67	0.111	0.739
Kyzylorda region	0.00	0.00	-	-		-
Mangystau region	0.26 (0.13–0.52)	0.44 (0.28–0.70)	−0.18 (−0.48 to 0.14)	69.23	1.555	0.212
Pavlodar region	0.04 (0.01–0.13)	0.25 (0.15–0.42)	−0.20 (−0.36 to −0.06)	525.00	8.906	0.003
North Kazakhstan region	0.39 (0.11–1.40)	0.00	-	−100.00	-	-
Turkestan region	0.00	0.00	-	-	-	-
Nationwide	0.20 (0.17–0.23)	0.18 (0.15–0.22)	0.01 (−0.03 to 0.05)	−10.00	0.363	0.547

## Data Availability

The datasets used and/or analyzed during the current study are available from the corresponding author upon reasonable request.

## Abbreviations

COVID-19	Coronavirus disease 2019
PAS	Psychoactive substances
RTAs	Road traffic accidents
NHTSA	National Highway Traffic Safety Administration
